# Direct anterior vs other surgical approaches in patients with lumbar stiffness undergoing total hip arthroplasty: a systematic review and meta-analysis

**DOI:** 10.1007/s00402-024-05682-y

**Published:** 2024-12-16

**Authors:** Liron Leibovitch, Elcio Machinski, André Fernandes, Jae Yong Park, Gabriel Souza, Iqbal F. Sayudo, Yaniv Warschawski, Caio Gusmao

**Affiliations:** 1 Department of Medicine, Azrieli Faculty of Medicine, Bar-Ilan University, Safed, Israel; 2 Department of Medicine, State University of Ponta Grossa, Ponta Grossa, Brazil; 3 Department of Orthopaedics and Trauma, York and Scarborough National Health Service Foundation Trust, York, UK; 4 Department of Medicine, Imperial College School of Medicine, London, UK; 5 Department of Medicine, Universidade Federal de Juiz de Fora, Juiz de Fora, Brazil; 6 Department of Medicine, Syiah Kuala University, Banda Aceh, Indonesia; 7 Department of Orthopaedics, Tel Aviv Sourasky Medical Center, Tel Aviv, Israel; 8 Department of Orthopaedics and Trauma, Hospital Santa Teresa, Petrópolis, Brazil

**Keywords:** Total hip arthroplasty, Direct anterior approach, Dislocations, Spinopelvic motion, Lumbar spine fusion

## Abstract

**Introduction:**

The effectiveness of the direct anterior approach (DAA) compared to other surgical approaches for total hip arthroplasty (THA) in patients with lumbar spine stiffness remains unclear. This systematic review and meta-analysis aimed to compare clinical outcomes, including dislocation rates and other complications, between DAA and other surgical approaches for THA in patients with lumbar spine stiffness.

**Materials and methods:**

We conducted a systematic review and meta-analysis to compare the DAA with other surgical approaches (anterolateral, direct lateral, posterolateral and direct superior) in patients with lumbar spine stiffness undergoing THA. We searched PubMed, Embase, and Cochrane Central databases for cohort studies and randomized controlled trials and calculated risk ratios (RRs) with 95% confidence intervals (CIs) to assess dislocation rates.

**Results:**

This analysis included 11 non-randomized studies comprising 2505 patients, of whom 738 patients (29.4%) underwent THA via DAA. The results demonstrated that the DAA group had significantly reduced dislocation rates (RR 0.31, 95% CI 0.14–0.67, P = 0.003, I^2^ = 0%) compared to other surgical approaches. Subgroup analysis showed significantly lower dislocation rates in DAA patients versus those undergoing the posterior approach (RR 0.22, 95% CI 0.10–0.52, P = 0.001, I^2^ = 0%). However, there was no statistically significant difference in dislocation rates between DAA and the lateral approach (RR 0.53, 95% CI 0.19–1.47, P = 0.22, I^2^ = 0%), although the rate was numerically lower.

**Conclusion:**

The DAA was associated with lower dislocation rates compared to other surgical techniques in patients with lumbar spine stiffness undergoing THA.

**Supplementary Information:**

The online version contains supplementary material available at 10.1007/s00402-024-05682-y.

## Introduction

Total hip arthroplasty (THA) is a reconstructive orthopaedic surgical procedure that replaces a damaged hip joint with an artificial prosthetic implant [1, 2] making it the preferred treatment for end-stage degenerative joint diseases [3]. Despite its high success rate [4], THA involves risks such as instability and dislocation, which depends on precise implant alignment, soft tissue management, and optimal spinopelvic balance [5]. Spinal stiffness significantly affects spinopelvic dynamics, increasing the surgical risks of acetabular malposition, impingement, and dislocations [6, 7].

The direct anterior approach (DAA), which utilizes a frontal hip incision navigating between the tensor fascia lata and the sartorius muscle, is noted for minimizing muscle damage. This approach facilitates improved prosthesis positioning and is associated with favorable long-term outcomes, implant success, and survivorship in the general population [8]. However, limited research has been conducted to compare the clinical outcomes of DAA with other THA approaches in patients with lumbar spine stiffness [9]. As a result, specific surgical guidelines tailored to this population are lacking [9]. We conducted the first systematic review and meta-analysis (SRMA) to assess the clinical outcomes of DAA compared to other THA approaches in patients with lumbar spine stiffness.

## Materials and methods

This SRMA adhered to the recommendations outlined in the Preferred Reporting Items for Systematic Reviews and Meta-Analyses (PRISMA) [10] statement and the Cochrane Handbook for Systematic Reviews of Interventions [11]. The study protocol was prospectively registered on PROSPERO (CRD42023479818). Institutional review board approval was not needed, given that only publicly available data from articles already published in the literature were used.

### Eligibility criteria

We included studies that were (1) randomized or nonrandomized longitudinal clinical studies; (2) comparing the DAA versus other THA approaches; (3) involving adult patients (aged 18 years or older) with lumbar spine stiffness; and (4) evaluating complication rates, functional outcomes, or radiographic outcomes. Exclusion criteria were as follows: (1) conference abstracts, review articles, and case reports; (2) studies where the surgical approach was not defined; and (3) studies where it is uncertain whether outcomes were specific to the DAA or other THA approaches. In cases of overlapping patient populations, the study with the largest sample size was included. A list of excluded studies with the reasons for exclusion is provided in the Supplemental Appendix. There were no restrictions on the publication date or language. Authors of excluded studies that did not report surgical approaches or outcomes by surgical approach were contacted to request the missing data.

### Search strategy

On September 21, 2024, we conducted a comprehensive literature search using PubMed, Cochrane, and Embase databases. The search strategy included terms related to total hip arthroplasty or hip replacement and associated abbreviations, lumbar spine stiffness and fusion procedures (e.g.: arthrodesis, lumbosacral fusion, lumbar fusion, spinal fusion, vertebrae fusion), and various anatomical and pathological terms related to lumbar spine conditions, such as lumbar deformity, spondylosis, and arthritis. The detailed search strategy for each database is outlined in the Supplementary Appendix. Additionally, we manually searched the reference lists of recent reviews and eligible studies to broaden our search and ensure comprehensive coverage.

### Data extraction

Following the removal of duplicates, two researchers (L. L. and E. M.) independently assessed the titles and abstracts of studies identified in the literature search. If a study was deemed potentially relevant, a full copy was obtained for a more detailed review to determine final inclusion based on the research question. Disagreements were resolved through consensus or, if necessary, via panel discussion with a third author (C. G.). We used a standardized tool to extract relevant information from the included studies, which included general study identification details such as authors' names, publication year, study period, country, and the number of patients. We also recorded baseline clinical characteristics, including sex, age, type of surgical approach, follow-up period, body mass index (BMI), type of bearing, number of fused levels, and whether the fusion involved the pelvis. Authors were contacted for additional information if it was unavailable in the original manuscript.

Outcomes of interest included complication rates (dislocation, aseptic loosening, infections, deep vein thrombosis, fractures, and revisions), post-operative functionality (evaluated using the Harris Hip Score, Japanese Orthopaedic Association score, and the Hip Disability and Osteoarthritis Outcome Score for Joint Replacement), length of stay, and radiographic assessments of prosthesis placement.

### Quality assessment

Two independent reviewers (L. L. and E. M.) assessed the risk of bias in the included studies using the Cochrane tool, Risk Of Bias In Non-randomized Studies of Interventions (ROBINS-I) [12]. Since our literature search yielded no randomized studies, the Risk of Bias 2 tool was not applicable. ROBINS-I evaluates studies across seven domains: confounding, selection, classification of interventions, deviations from intended interventions, missing data, measurement of outcomes, and selection of reported results. Each domain is rated as low risk, moderate risk, serious risk, critical risk, or no information. Studies are scored based on the highest risk of bias identified across domains. Disagreements were resolved through consensus or, if necessary, by consulting a third author (C. G.). Publication bias was investigated using funnel plot inspection of point estimates according to study weights and Egger's regression test.

### Statistical analysis

We compared pooled effects for binary outcomes using risk ratios (RR) and for continuous endpoints using mean differences (MD), both accompanied by 95% confidence intervals (CI). The Mantel–Haenszel random effects model was utilized for all analyses to account for expected between-study variability in study methods and population, independently of heterogeneity measures. Heterogeneity was assessed using the Cochran Q test and I^2^ statistics, with P values below 0.10 and I^2^ over 25% indicating significant heterogeneity. Additionally, a sensitivity analysis employing the generic inverse variance method was conducted to assess the robustness of our findings. Statistical significance was established at a P value of less than 0.05. All statistical analyses were performed using Review Manager 5.4.1 (Cochrane Collaboration, Copenhagen, Denmark) and R version 4.3.2 (R Foundation, Vienna, Austria).

## Results

### Study selection and characteristics

**Fig. 1:**
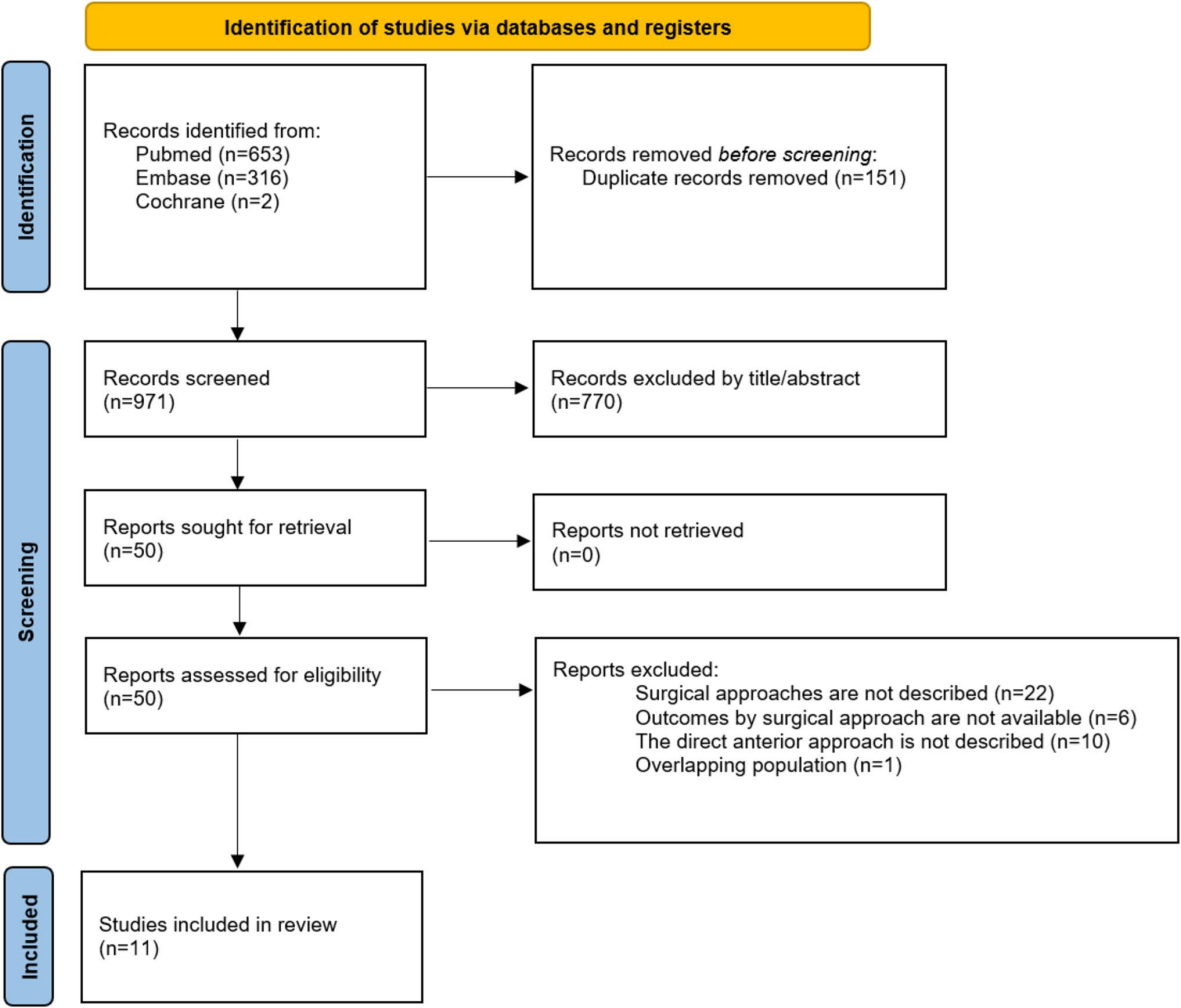
PRISMA flow diagram depicting the study selection process

A total of 971 studies were identified through database searches and manual review of references from included studies and reviews (Fig. 1). After removing duplicates and unrelated publications, 50 articles underwent full review based on inclusion and exclusion criteria. Ultimately, 11 non-randomized studies involving 2505 patients were included, with 738 patients (29.4%) undergoing THA via DAA.

Clinical results of the DAA were compared with the lateral approach (anterolateral and direct lateral) in nine studies and with the posterior approach (posterolateral, superior direct) in ten studies. The proportion of surgical approaches in the control group were posterior approach, 921 patients (36.7%); lateral approach, 332 patients (13.2%); anterolateral approach, 214 patients (8.5%); superior direct approach, 4 patients (0.2%); and other approaches, 15 patients (0.6%). Across the studies, male sex prevalence ranged from 24.0 to 50.5%, median age varied from 64.0 to 71.0 years, median BMI ranged from 25.0 to 33.0 kg/m^2^, and mean follow-up varied from 1 to 18 years. The baseline characteristics of the included studies are summarized in Table 1.

**Table 1:** Baseline characteristics of included studies

Studies	Arms and approaches	Dislocation rate	Age, years	Male sex	BMI, kg/m^2^	No. levels fused	Spine fused to pelvis	Bearing type	Follow up, years
Andah, 2021 (N = 67)	DA, 18 (27) L, 18 (27) P, 31 (46)	1 (6) 1 (6) 6 (19)	68 (NR)	19 (28)	29 (NR)	NR^a^	NR	NR	1 (NR)
Salib, 2019 (N = 97)	DA, 9 (9) AL, 49 (51) PL, 39 (40)	0 1 (2) 4 (10)	71 (40–87)	43 (44)	30 (19–45)	1, 43 (44) ≥ 2, 21 (22)	33 (34)	F, 97 (100)	6 (2–17)
Ochiai, 2023 (N = 67)	DA, 27 (40) AL, 18 (27) PL, 22 (33)	0 3 (17) 2 (9)	64 (43–80)	16 (24)	25 (NR)	1–2, 46 (69) 3–6, 10 (15) ≥ 7, 11 (16)	NR	NR	3 (1–17)
Nessler, 2020 (N = 93)	DA, 11 (12) P, 63 (68) DL, 15 (16) DS, 4 (4)	0 0 0 0	65 (46–87)	37 (39)	30 (18–48)	1, 41 (44) 2, 27 (29) ≥ 3, 14 (15)	NR	DM, 93 (100)	3 (1–10)
Di Martino, 2023 (N = 547)	DA, 79 (14) L, 281 (51) PL, 172 (31) Nonspecified, 15 (2)	0 3 (1) 5 (3) 0	68 (30–91)^d^	208 (38)	NR^e^	NR	NR	S, 547 (96) DM, 21 (4)	Up to 18^f^
Khan, 2023 (N = 208)	DA, 83 (40) AL, 71 (34) DL, 21 (10) PL, 33 (16)	0 0 0 0	1 LSF, 67 (45–88) 2 LSF, 68 (53–81) ≥ 3 LSF, 67 (51–78)	1 LSF, 41 (39) 2 LSF, 12 (22) ≥ 3 LSF, 24 (50)	1 LSF, 30 (5) 2 LSF, 29 (6) ≥ 3 LSF, 29 (5)	1, 105 (50) 2, 55 (26) ≥ 3, 48 (23)	4 (2)	NR	At least 1
Goyal, 2022 (N = 332)	DA, 89 (27) DL, 243 (73)	1 (1) 2 (1)	DA, 65 (63–67) DL, 67 (65–68)	DA, 37 (42) DL, 84 (35)	DA, 31 (29–32) DL, 30 (28–30)	1: DA, 40 (45); DL, 80 (33) 2: DA, 22 (25); DL, 76 (33) ≥ 3: DA, 27 (30); DL, 87 (36)	DA, 16 (18) DL, 214 (88)	S, 332 (100)	At least 1
Minutillo, 2023 (N = 111)	DA, 37 (33) P, 74 (67)	0 4 (5)	DA, 68 (NR) P, 68 (NR)	DA, 10 (27) P, 22 (30)	DA, 26 (NR) P, 33 (NR)	NR^b^	DA, 24 (65) P, 32 (43)	CoP: DA, 21 (56); P, 66 (89) DM: DA, 16 (43); P, 8 (11)	11 (NR)
Sarpong, 2023 (N = 367)	DA, 181 (49) PL, 186 (51)	1 (1) 5 (3)	DA, 66 (36–91) PL, 67 (18–89)	DA, 73 (40) PL, 91 (49)	DA, 26 (17–39) PL, 30 (16–53)	1–2: DA, 7 (4); PL, 7 (4) ≥ 3: DA, 4 (2); PL, 5 (3)	DA, 6 (4) PL, 6 (4)	NR	2 (NR)
Iturregui, 2023 (N = 293)	DA, 117 (40) AL, 76 (26) P, 101 (34)	1 (1) 4 (5) 4 (4)	NR	DA, 62 (53) AL, 35 (46) P, 51 (50)	DA, 29 (NR) AL, 29 (NR) P, 31 (NR)	NR^c^	NR	NR^g^	DA, 3 (NR) AL, 4 (NR) P, 4 (NR)
Huebschmann, 2024 (N = 323)	DA, 87 (27) L, 36 (11) P, 200 (62)	1 (1) 0 14 (7)	66 (22–88)	130 (40)	31 (18–52)	NR^h^	DA, 45 (51) L, 17 (47) P, 100 (50)	S, 5 (33)^i^ L, 6 (40)^i^ DM, 4 (27)^i^	NR

Values are presented as n (%) or median (range). Percentages may not sum to 100% due to rounding. Unless specified, values represent the entire cohort

*AL* Anterolateral, *CoP* ceramic on polyethylene, *DA* Direct anterior, *DL* Direct lateral, *DM* Dual Mobility, *DS* Direct superior, *L* lateral, *LSF* lumbar spine fusion, *NR* not reported, *PL* posterolateral, *P* Posterior

^a^Median and range: 2.4 (1–16)

^b^Mean and standard deviation: 2.4 (2)

^c^Mean and standard deviation: 2.4 (2)

^d^Average (min–max)

^e^Average weight (standard deviation): 76 (14)

^f^Approximate values

^g^Lipped liners and dual mobility components were not routinely used

^h^Mean (range) of number of levels fused: 3 (1–23)

^i^Bearing type collected only for patients with dislocation [n = 15 (4)]

### Dislocation rates

**Fig. 2:**
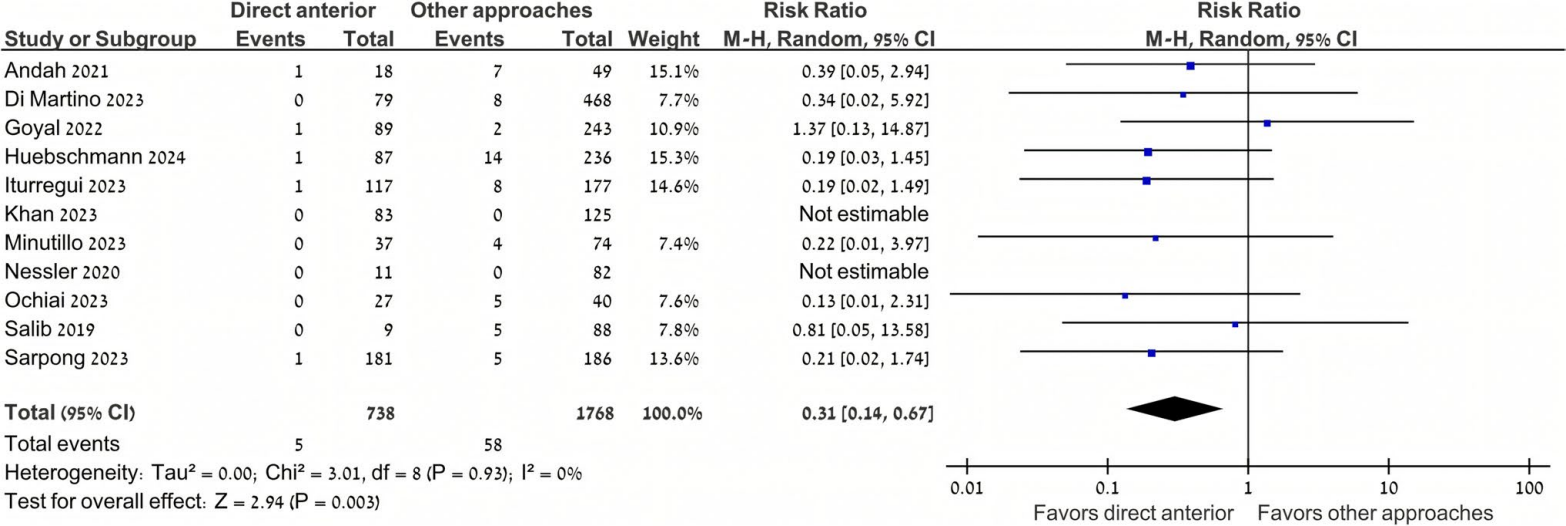
Dislocation rate was significantly lower in the direct anterior approach

**Fig. 3:**
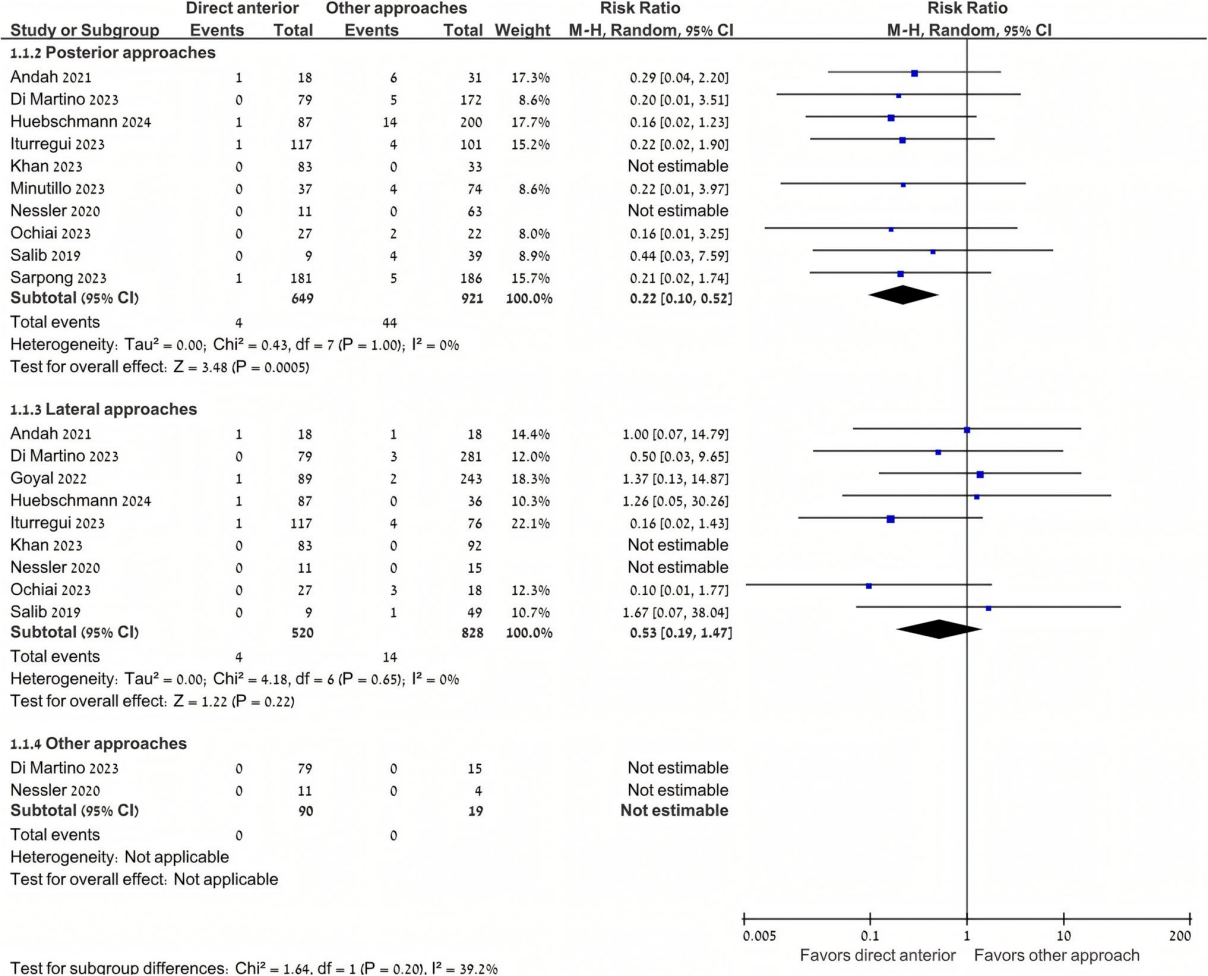
Dislocation rates were lower with the direct anterior approach compared to the posterior approach. Compared with posterior approaches, the direct anterior approach was associated with a lower rate of dislocations (P < 0.001). However, compared with lateral approaches, lower rates in the direct anterior approach failed to meet statistical significance (P = 0.22). There were no comparisons between direct anterior and other approaches. There was no interaction between comparisons of direct anterior with lateral or posterior approaches

Patients undergoing DAA had significantly lower dislocation rates (0.7% [5/738]) compared to those undergoing other THA approaches (3.3% [58/1768]) (RR 0.31, 95% CI 0.14–0.67, P = 0.003, I^2^ = 0%) (Fig. 2). Subgroup analyses showed lower dislocation rates among patients undergoing DAA (0.6% [4/649]) as compared with the posterior approach (4.8% [44/921]) (RR 0.22, 95% CI 0.10–0.52, P < 0.001, I^2^ = 0%) (Fig. 3). However, differences between DAA (0.8% [4/520]) and the lateral approach (1.7% [14/828]) failed to meet statistical significance (RR 0.53, 95% CI 0.19–1.47, P = 0.22, I^2^ = 0%) (Fig. 3). An exploratory analysis showed no significant treatment interaction in the effect size of the comparison between DAA vs. the lateral and posterior approaches for THA in this population (P = 0.20) (Fig. 3).

### Other outcomes

Outcomes other than dislocation could not be pooled in meta-analyses due to significant heterogeneity in definitions and assessment methods (Supplemental Table 1). Regarding post-operative functionality, six studies indicated enhancements in pre- and post-operative functionality scores in DAA groups [13–18]. Two studies [13, 14] demonstrated improved scores across patients undergoing DAA: the first [13] reported a mean increase of 31 points in the Harris Hip Score, while the second [14] observed a 34-point increase in the mean Japanese Orthopaedic Association score. In two studies [16, 18], patients undergoing DAA experience a shorter hospital stay of one day.

Considerable variation was observed among seven studies [13–15, 19–22] regarding complication outcomes other than dislocations, such as aseptic loosening, infections, deep vein thrombosis, fractures, and revisions. One study [20] reported comparable revision rates between DAA and alternative approaches. However, another study [21] identified lower revision frequencies associated with DAA. Incidences of aseptic loosening and infection did not significantly differ across surgical approaches, with most studies reporting rates as low as 0% [13–15, 22].

### Radiographic assessment

The report of postoperative radiographic assessments in the included studies was not consistently stratified between the surgical arms. Therefore, we report these results as a systematic review of available data, without a pooled analysis of outcomes (Supplementary Table 1). In one study [23], anteversion angles increased by 2° for DAA and 3° for the direct lateral approach (P = 0.57). Additionally, post-surgery, there was a 4° increase in sacral slope with DAA and a 1° increase with the direct lateral approach post-surgery (P = 0.60). Furthermore, both approaches led to a 3° rise in lumbar lordosis (P = 0.74). In the same study, patients who underwent DAA had a 6° increase in pelvic incidence-lumbar lordosis mismatch, while a 5° reduction was observed in those who underwent the direct lateral approach (P = 0.29). Meanwhile, pelvic tilt remained stable after DAA but decreased by 1° post-direct lateral surgery (P = 0.33).

### Sensitivity analysis

To reinforce the robustness of our findings, we conducted a sensitivity analysis which yielded results consistent with the primary analyses. DAA was associated with lower dislocation rates compared to all other THA approaches (RR 0.33, 95% CI 0.14–0.78, P = 0.01, I^2^ = 0%) (Supplemental Fig. 1), and when compared to the posterior approach (RR 0.24, 95% CI 0.09–0.60, P = 0.03, I^2^ = 0%) (Supplemental Fig. 2). No differences in dislocation rates were observed when comparing DAA to the lateral approach (RR 0.48, 95% CI 0.16–1.41, P = 0.18, I^2^ = 0%) (Supplemental Fig. 2).

### Quality assessment

ROBINS-I assessments indicated that all included studies lacked sufficient adjustment for confounding (Supplemental Table 2). Out of the 11 studies reviewed, 4 showed a moderate overall risk of bias from confounding factors, while 7 exhibited a serious overall risk of bias. The overall bias was solely due to factors in the confounding domain, while all other domains were assessed as low risk. There was no evidence of publication bias, as demonstrated by both funnel plot inspection (Supplemental Fig. 3) and Egger’s regression test (P = 0.63).

## Discussion

In this SRMA of 2,505 patients across 11 studies, we evaluated the outcomes of the DAA compared to other surgical approaches for THA in patients with lumbar spine stiffness. Our analysis revealed that DAA is associated with lower dislocation rates compared to alternative approaches. This study is the first to systematically review and analyze surgical outcomes across different THA approaches in this patient demographic. Due to inconsistent reporting and lack of standardization in assessing other outcomes, our analysis was limited to a systematic review covering functionality, other perioperative complications, and radiographic measurements. The variability in reporting these outcomes poses challenges in accurately interpreting and comparing the effectiveness of the surgical approaches.

DAA is well-recognized for its protective benefits in THA [8, 24]. This approach minimizes damage to posterior soft tissues and preserves the posterior hip capsule by utilizing muscle-sparing dissection within an internervous plane [25]. These features are crucial in reducing postoperative complications, especially dislocations, by preserving the integrity of key stabilizing structures [26].

In subgroup analyses comparing different surgical approaches, DAA was significantly associated with a lower risk of dislocation compared to the posterior approach, but not when compared to the lateral approach. However, there was no significant treatment interaction in the effect of DAA vs. posterior or lateral approach. This suggests the benefit of DAA on lowering dislocation rates may extend across different surgical approaches. The absence of statistical significance in the comparison to the lateral approach is likely due to insufficient statistical power.

The optimization of cup anteversion during THA with the DAA may contribute to the favorable results in this group. Unlike the lateral decubitus positioning used in the posterior approach, the anterior and lateral approaches utilize supine positioning. This alignment allows for more precise adjustments of the cup's anteversion angle, aligning closely with the natural alignment of the pelvis [9]. This precise positioning may account for the smaller numerical differences in dislocation rates between DAA and the lateral approach compared to those between DAA and the posterior approach.

Additionally, DAA potentially offers protective benefits for patients with lumbar spine stiffness, a condition linked to higher risks of surgical complications, particularly dislocations. The supine positioning inherent to DAA not only facilitates greater hip stability through accurate cup positioning, but also preserves more soft tissue. These advantages collectively support less restricted, faster, and safer rehabilitation for patients with lumbar spine stiffness undergoing THA.

As the population ages, the number of individuals with lumbar spine stiffness requiring THA is expected to increase [27], underscoring the need to identify the most effective surgical approach. Our meta-analysis suggests that DAA could be considered a preferred approach for THA in patients with lumbar spine stiffness. However, further randomized controlled trials are necessary to confirm its effectiveness with greater certainty.

### Limitations

Our study has limitations. First, the lack of randomized controlled trials hinders our ability to infer causality definitively. While other methodological aspects exhibited a low risk of bias, the retrospective cohort design of the included studies introduces serious concerns regarding control for confounding and inherent biases, which could compromise the validity of our conclusions.

A key limitation of our analysis was the inconsistent and heterogeneous reporting of surgical-related variables across studies. The utilization of computer-assisted surgery techniques, including CT-based navigation, was not uniformly reported. These advanced technologies, which can be associated with various surgical approaches including DAA, may independently influence outcomes. The lack of consistent information prevented us from conducting subgroup analyses to isolate the effects of these techniques from those of the surgical approach itself. This limitation highlights the need for more standardized reporting in future studies to better understand the interplay between surgical approach and advanced technologies.

Furthermore, the variability in implant types, particularly the use of standard versus dual-mobility bearings, presents another potential confounding factor. While dual-mobility implants were infrequently used in our cohort, with only one study [15] reporting 100% usage, the impact of implant choice on dislocation rates cannot be definitively determined from our data. Future research should aim to control for these variables more rigorously to provide clearer insights into the independent effects of surgical approach on outcomes.

The significant heterogeneity observed across the studies in methodologies, patient characteristics, and outcome definitions restricted our ability to conduct meta-analyses for outcomes other than dislocations. However, this heterogeneity did not substantially affect the between-group differences in dislocation rates, and our findings remained robust across various statistical methods, thereby enhancing the reliability of our results. Nevertheless, further studies addressing these limitations are warranted.

## Conclusion

This SRMA suggests that DAA is associated with lower dislocation rates in patients with lumbar spine stiffness undergoing THA compared to other surgical techniques. However, this finding should be interpreted cautiously due to study limitations. Further research, particularly randomized controlled trials, is needed to confirm these results and explore other outcomes.

## Supplementary Information

Below is the link to the electronic supplementary material.

Supplementary file1 (DOCX 479 KB)

## Data Availability

All data analyzed in this meta-analysis were extracted from previously published studies, which are listed in our references section.

## Abbreviations

AL: Anterolateral
BMI: Body mass index
CI: Confidence intervals
CoP: Ceramic on polyethylene
DAA: Direct anterior approach
DL: Direct lateral
DM: Dual Mobility
DS: Direct superior
LSF: Lumbar spine fusion
MD: Mean difference
NA: Not applicable
NR: Not reported
PL: Posterolateral
PRISMA: Preferred Reporting Items for Systematic Reviews and Meta-Analysis
ROBINS-I: Risk of Bias in Non-Randomized Studies
RR: Risk ratios
SRMA: Systematic Review and Meta-Analysis
THA: Total hip arthroplasty
