# Supplementary material for: Direct anterior vs other surgical approaches in patients with lumbar stiffness undergoing total hip arthroplasty: a systematic review and meta-analysis
Source: Arch Orthop Trauma Surg. 2024 Dec 16;145(1):48. doi: 10.1007/s00402-024-05682-y (PMC11649716; doi:10.1007/s00402-024-05682-y)
Supplement: Supplementary file 1 — Supplementary file1 (DOCX 479 KB) [file 402_2024_5682_MOESM1_ESM.docx]

**Supplemental Material**

**Direct Anterior or Other Surgical Approaches in Patients with Lumbar Stiffness Undergoing Total Hip Arthroplasty:**

A Systematic Review and Meta-Analysis

**Content**

Full search string….……………………………………………………………………………...2

Reasons for exclusion after full-text review...………………………………………………...….3

PRISMA 2020 checklist ………………………………………………………………….…...…8

Supplementary Table 1……………………………………………………………………….…11

Supplementary Table 2……………………………………………………………………….…13

Supplementary Figures………………………………………………………………………….14

**Full search string**

- PubMed

("Total Hip Arthroplasty" OR "Hip Replacement" OR "Hip Arthroplasty" OR "Hip Joint Replacement" OR "Hip Joint Arthroplasty" OR THA OR THR OR "Arthroplasty, Replacement, Hip"[Mesh] OR "Robotic-Assisted Hip Arthroplasty" OR "Computer-Assisted Hip Arthroplasty" OR "Navigation-Assisted Hip Arthroplasty" OR "Dual Mobility Hip Arthroplasty") AND ("Lumbar Fusion" OR "Spinal Fusion" OR "Spine Fusion" OR "Lumbar Vertebrae Fusion" OR "Back Fusion" OR "Lumbar Spondylodesis" OR "Lumbar Interbody Fusion" OR Arthrodesis OR "Lumbosacral Fusion" OR LSF OR PLF OR ALF OR LIF OR "Spinal Fusion"[Mesh] OR "Lumbar spinal deformity" OR Spondylosis OR "Lumbar Spine Degeneration" OR "Lumbar Arthritis" OR "Lumbosacral Osteoarthritis" OR "Spinal Fusion"[Mesh] OR "Spondylosis"[Mesh])

- Embase

("Total Hip Arthroplasty" OR "Hip Replacement" OR "Hip Arthroplasty" OR "Hip Joint Replacement" OR "Hip Joint Arthroplasty" OR THA OR THR ) AND ("Lumbar Fusion" OR "Spinal Fusion" OR "Spine Fusion" OR "Lumbar Vertebrae Fusion" OR "Back Fusion" OR "Lumbar Spondylodesis" OR "Lumbar Interbody Fusion" OR Arthrodesis OR "Lumbosacral Fusion" OR LSF OR PLF OR ALF OR LIF OR "Lumbar spinal deformity" OR Spondylosis OR "Lumbar Spine Degeneration" OR "Lumbar Arthritis" OR "Lumbosacral Osteoarthritis" )

- Cochrane Library

("Total Hip Arthroplasty" OR "Hip Replacement" OR "Hip Arthroplasty" OR "Hip Joint Replacement" OR "Hip Joint Arthroplasty" OR THA OR THR ) AND ("Lumbar Fusion" OR "Spinal Fusion" OR "Spine Fusion" OR "Lumbar Vertebrae Fusion" OR "Back Fusion" OR "Lumbar Spondylodesis" OR "Lumbar Interbody Fusion" OR Arthrodesis OR "Lumbosacral Fusion" OR LSF OR PLF OR ALF OR LIF OR "Lumbar spinal deformity" OR Spondylosis OR "Lumbar Spine Degeneration" OR "Lumbar Arthritis" OR "Lumbosacral Osteoarthritis" )

**Reasons for exclusion after full-text review**

- Surgical approaches are not described
  - Mohamed, N. S., Salib, C. G., Sax, O. C., Remily, E. A., Douglas, S. J., & Delanois, R. E. (2024). Spinal fusion and total hip arthroplasty: why timing is important. Hip international : the journal of clinical and experimental research on hip pathology and therapy, 34(2), 174–180. <https://doi.org/10.1177/11207000231197420>.
  - Bala A, Chona DV, Amanatullah DF, et al. Timing of Lumbar Spinal Fusion Affects Total Hip Arthroplasty Outcomes. J Am Acad Orthop Surg Glob Res Rev. 2019;3(11):e00133. Published 2019 Nov 4. doi:10.5435/JAAOSGlobal-D-19-00133
  - Klemt C, Padmanabha A, Tirumala V, Walker P, Smith EJ, Kwon YM. Lumbar Spine Fusion Before Revision Total Hip Arthroplasty Is Associated With Increased Dislocation Rates. J Am Acad Orthop Surg. 2021;29(17):e860-e868. doi:10.5435/JAAOS-D-20-00824
  - Zhang SE, Anatone AJ, Figgie MP, Long WJ, Della Valle AG, Lee GC. Spine or Hip First? Outcomes in Patients Undergoing Sequential Lumbar Spine or Hip Surgery. J Arthroplasty. 2023;38(7S):S114-S118.e2. doi:10.1016/j.arth.2023.04.030
  - Malkani AL, Garber AT, Ong KL, et al. Total Hip Arthroplasty in Patients With Previous Lumbar Fusion Surgery: Are There More Dislocations and Revisions?. J Arthroplasty. 2018;33(4):1189-1193. doi:10.1016/j.arth.2017.10.041
  - Bedard NA, Martin CT, Slaven SE, Pugely AJ, Mendoza-Lattes SA, Callaghan JJ. Abnormally High Dislocation Rates of Total Hip Arthroplasty After Spinal Deformity Surgery. J Arthroplasty. 2016;31(12):2884-2885. doi:10.1016/j.arth.2016.07.049
  - Ramamurti P, Quinlan ND, Deasey MJ, Kamalapathy PN, Werner BC, Browne JA. Increased Dislocation Rates After Total Hip Arthroplasty in Patients With Prior Isolated Sacroiliac Joint Arthrodesis. J Arthroplasty. 2023;38(7S):S101-S105.e1. doi:10.1016/j.arth.2023.03.051
  - Barry JJ, Sing DC, Vail TP, Hansen EN. Early Outcomes of Primary Total Hip Arthroplasty After Prior Lumbar Spinal Fusion. J Arthroplasty. 2017;32(2):470-474. doi:10.1016/j.arth.2016.07.019
  - Blizzard DJ, Sheets CZ, Seyler TM, et al. The Impact of Lumbar Spine Disease and Deformity on Total Hip Arthroplasty Outcomes. Orthopedics. 2017;40(3):e520-e525. doi:10.3928/01477447-20170327-03
  - Welling S, Smith S, Yoo J, Philipp T, Mildren M, Kagan R. Is Timing of Total Hip Arthroplasty and Lumbar Spine Fusion Associated With Risk of Hip Dislocation?. Arthroplast Today. 2023;23:101202. Published 2023 Sep 16. doi:10.1016/j.artd.2023.101202
  - Murphy MP, Schneider AM, LeDuc RC, Killen CJ, Adams WH, Brown NM. A Multivariate Analysis to Predict Total Hip Arthroplasty Dislocation With Preoperative Diagnosis, Surgical Approach, Spinal Pathology, Cup Orientation, and Head Size. J Arthroplasty. 2022;37(1):168-175. doi:10.1016/j.arth.2021.08.031
  - Sing DC, Barry JJ, Aguilar TU, et al. Prior Lumbar Spinal Arthrodesis Increases Risk of Prosthetic-Related Complication in Total Hip Arthroplasty. J Arthroplasty. 2016;31(9 Suppl):227-232.e1. doi:10.1016/j.arth.2016.02.069
  - Malkani AL, Himschoot KJ, Ong KL, et al. Does Timing of Primary Total Hip Arthroplasty Prior to or After Lumbar Spine Fusion Have an Effect on Dislocation and Revision Rates?. J Arthroplasty. 2019;34(5):907-911. doi:10.1016/j.arth.2019.01.009
  - Di Martino A, Bordini B, Geraci G, et al. Impact of previous lumbar spine surgery on total hip arthroplasty and vice versa: How long should we be concerned about mechanical failure?. Eur Spine J. 2023;32(9):2949-2958. doi:10.1007/s00586-023-07866-3
  - Nessler JM, Malkani AL, Yep PJ, Mullen KJ, Illgen RL 2nd. Dislocation Rates of Primary Total Hip Arthroplasty in Patients With Prior Lumbar Spine Fusion and Lumbar Degenerative Disk Disease With and Without Utilization of Dual Mobility Cups: An American Joint Replacement Registry Study. J Am Acad Orthop Surg. 2023;31(5):e271-e277. doi:10.5435/JAAOS-D-22-00767
  - Buckland AJ, Puvanesarajah V, Vigdorchik J, et al. Dislocation of a primary total hip arthroplasty is more common in patients with a lumbar spinal fusion. Bone Joint J. 2017;99-B(5):585-591. doi:10.1302/0301-620X.99B5.BJJ-2016-0657.R1
  - Yang DS, Li NY, Mariorenzi MC, Kleinhenz DT, Cohen EM, Daniels AH. Surgical Treatment of Patients With Dual Hip and Spinal Degenerative Disease: Effect of Surgical Sequence of Spinal Fusion and Total Hip Arthroplasty on Postoperative Complications. Spine (Phila Pa 1976). 2020;45(10):E587-E593. doi:10.1097/BRS.0000000000003351
  - Kagan R, Welling S, Mildren ME, Smith S, Philipp T, Yoo J. It is the Opioids Not the Spine Surgeon; Dislocation After Total Hip Arthroplasty is Associated With Opioid Use in Patients Who Have Prior Lumbar Spine Fusion. J Arthroplasty. 2023;38(7 Suppl 2):S336-S339. doi:10.1016/j.arth.2023.02.080
  - Yang DS, McDonald CL, DiSilvestro KJ, et al. Risk of Dislocation and Revision Following Primary Total Hip Arthroplasty in Patients With Prior Lumbar Fusion With Spinopelvic Fixation. J Arthroplasty. 2023;38(4):700-705.e1. doi:10.1016/j.arth.2022.03.061
  - Ofa SA, Lupica GM, Lee OC, Sherman WF. Complications following total hip arthroplasty and hemiarthroplasty for femoral neck fractures in patients with a history of lumbar spinal fusion. Arch Orthop Trauma Surg. 2023;143(2):817-827. doi:10.1007/s00402-021-04158-7
  - Blizzard DJ, et al. Lumbar spine disease negatively affects outcomes after total hip arthroplasty. Spine J. 2015;15(10):S154.
  - Diebo BG, Beyer GA, Grieco PW, et al. Complications in Patients Undergoing Spinal Fusion After THA. Clin Orthop Relat Res. 2018;476(2):412-417. doi:10.1007/s11999.0000000000000009
- Outcomes by surgical approach are not available
  - Barry JJ, Sing DC, Vail TP, Hansen EN. Early Outcomes of Primary Total Hip Arthroplasty After Prior Lumbar Spinal Fusion. J Arthroplasty. 2017;32(2):470-474. doi:10.1016/j.arth.2016.07.019
  - Katakam A, Bedair HS, Melnic CM. Do All Rigid and Unbalanced Spines Present the Same Risk of Dislocation After Total Hip Arthroplasty? A Comparison Study Between Patients With Ankylosing Spondylitis and History of Spinal Fusion. J Arthroplasty. 2020;35(12):3594-3600. doi:10.1016/j.arth.2020.06.048
  - Hinman AD, Inacio MCS, Prentice HA, et al. Lumbar Spine Fusion Patients See Similar Improvements in Physical Activity Level to Non-Spine Fusion Patients Following Total Hip Arthroplasty. J Arthroplasty. 2020;35(2):451-456. doi:10.1016/j.arth.2019.08.053
  - Ameztoy Gallego J, Cruz Pardos A, Gomez Luque J, Cuadrado Rubio LE, Fernández Fernández R. Dislocation and survival rate of dual mobility cups in revision total hip arthroplasty in patients with high risk of instability. Int Orthop. 2023;47(7):1757-1764. doi:10.1007/s00264-023-05816-8
  - Grammatopoulos G, Gofton W, Jibri Z, et al. 2018 Frank Stinchfield Award: Spinopelvic Hypermobility Is Associated With an Inferior Outcome After THA: Examining the Effect of Spinal Arthrodesis. Clin Orthop Relat Res. 2019;477(2):310-321. doi:10.1097/CORR.0000000000000367
  - Loh JLM, Jiang L, Chong HC, Yeo SJ, Lo NN. Effect of Spinal Fusion Surgery on Total Hip Arthroplasty Outcomes: A Matched Comparison Study. J Arthroplasty. 2017;32(8):2457-2461. doi:10.1016/j.arth.2017.03.031
- The direct anterior approach is not described
  - Parilla FW, Shah RR, Gordon AC, et al. Does It Matter: Total Hip Arthroplasty or Lumbar Spinal Fusion First? Preoperative Sagittal Spinopelvic Measurements Guide Patient-Specific Surgical Strategies in Patients Requiring Both. J Arthroplasty. 2019;34(11):2652-2662. doi:10.1016/j.arth.2019.05.053
  - Guan H, Xu C, Fu J, Yang X, Zhang Y, Chen J. Ankylosing Spondylitis Patients Have Lower Risk of Dislocation Following Total Hip Arthroplasty Compared with Patients Undergoing Lumbar Spinal Fusion Surgery. Int J Gen Med. 2022;15:6573-6582. Published 2022 Aug 11. doi:10.2147/IJGM.S373432
  - Inoue D, Kabata T, Kajino Y, et al. The influence of surgical approach on postoperative pelvic tilt after total hip arthroplasty. Eur J Orthop Surg Traumatol. 2017;27(8):1131-1138. doi:10.1007/s00590-017-1946-4
  - Furuhashi H, Yamato Y, Hoshino H, et al. Dislocation rate and its risk factors in total hip arthroplasty with concurrent extensive spinal corrective fusion with pelvic fixation for adult spinal deformity. Eur J Orthop Surg Traumatol. 2021;31(2):283-290. doi:10.1007/s00590-020-02764-6
  - Fessy MH, Putman S, Viste A, et al. What are the risk factors for dislocation in primary total hip arthroplasty? A multicenter case-control study of 128 unstable and 438 stable hips [published correction appears in Orthop Traumatol Surg Res. 2017 Nov;103(7):1137]. Orthop Traumatol Surg Res. 2017;103(5):663-668. doi:10.1016/j.otsr.2017.05.014
  - Graf R, Azizbaig-Mohajer M. Minimally invasive total hip replacement with the patient in the supine position and the contralateral leg elevated. Oper Orthop Traumatol. 2006;18(4):317-329. doi:10.1007/s00064-006-1180-4
  - DelSole EM, Vigdorchik JM, Schwarzkopf R, Errico TJ, Buckland AJ. Total Hip Arthroplasty in the Spinal Deformity Population: Does Degree of Sagittal Deformity Affect Rates of Safe Zone Placement, Instability, or Revision?. J Arthroplasty. 2017;32(6):1910-1917. doi:10.1016/j.arth.2016.12.039
  - Guo Zhuotao, Zhang Kai, Zha Guochun, Guo Kaijin. A matched controlled trial of lumbar fusion effect on mid-term outcomes after total hip arthroplasty[J]. Chinese Journal of Tissue Engineering Research, 2023, 27(36): 5801-5805.
  - Huerfano, Elina MDa; Alzate, Ricardo MDa; Muñoz, Juan M. MDa; Riveros, Emilio A. MDb; Márquez, Diego MDb; Páez, Rodolfo MDb; Nossa, Juan M. MDa,b. Considerations in spinopelvic parameters and acetabular component orientation in patients with lumbar spinal fusion following total hip arthroplasty: A retrospective study. Current Orthopaedic Practice 31(3):p 218-223, May/June 2020. | DOI: 10.1097/BCO.0000000000000872
- Overlapping population
  - Di Martino A, Bordini B, Ancarani C, Viceconti M, Faldini C. Does total hip arthroplasty have a higher risk of failure in patients who undergo lumbar spinal fusion?. Bone Joint J. 2021;103-B(3):486-491. doi:10.1302/0301-620X.103B3.BJJ-2020-1209.R1

|  | | | |
| --- | --- | --- | --- |
| **PRISMA 2020 Checklist** | | | |
| **Section and Topic** | **Item #** | **Checklist item** | **Location where item is reported** |
| **TITLE** | | |  |
| Title | 1 | Identify the report as a systematic review. | Line 1-3 |
| **ABSTRACT** | | |  |
| Abstract | 2 | See the PRISMA 2020 for Abstracts checklist. | Not Applicable |
| **INTRODUCTION** | | |  |
| Rationale | 3 | Describe the rationale for the review in the context of existing knowledge. | Line 133-136 |
| Objectives | 4 | Provide an explicit statement of the objective(s) or question(s) the review addresses. | Line 136-138 |
| **METHODS** | | |  |
| Eligibility criteria | 5 | Specify the inclusion and exclusion criteria for the review and how studies were grouped for the syntheses. | Line 150-160 |
| Information sources | 6 | Specify all databases, registers, websites, organisations, reference lists and other sources searched or consulted to identify studies. Specify the date when each source was last searched or consulted. | Line 163-164, 169-171 |
| Search strategy | 7 | Present the full search strategies for all databases, registers and websites, including any filters and limits used. | Line 163-171 |
| Selection process | 8 | Specify the methods used to decide whether a study met the inclusion criteria of the review, including how many reviewers screened each record and each report retrieved, whether they worked independently, and if applicable, details of automation tools used in the process. | Line 174-178 |
| Data collection process | 9 | Specify the methods used to collect data from reports, including how many reviewers collected data from each report, whether they worked independently, any processes for obtaining or confirming data from study investigators, and if applicable, details of automation tools used in the process. | Line 174-184 |
| Data items | 10a | List and define all outcomes for which data were sought. Specify whether all results that were compatible with each outcome domain in each study were sought (e.g. for all measures, time points, analyses), and if not, the methods used to decide which results to collect. | Line 178-189 |
|  | 10b | List and define all other variables for which data were sought (e.g. participant and intervention characteristics, funding sources). Describe any assumptions made about any missing or unclear information. | Line 178-189 |
| Study risk of bias assessment | 11 | Specify the methods used to assess risk of bias in the included studies, including details of the tool(s) used, how many reviewers assessed each study and whether they worked independently, and if applicable, details of automation tools used in the process. | Line 192-201 |
| Effect measures | 12 | Specify for each outcome the effect measure(s) (e.g. risk ratio, mean difference) used in the synthesis or presentation of results. | Line 204-206 |
| Synthesis methods | 13a | Describe the processes used to decide which studies were eligible for each synthesis (e.g. tabulating the study intervention characteristics and comparing against the planned groups for each synthesis (item #5)). | Line 150-153, 174-177, 178-181 |
|  | 13b | Describe any methods required to prepare the data for presentation or synthesis, such as handling of missing summary statistics, or data conversions. | Line 183-184, 204-206, 208-209 |
|  | 13c | Describe any methods used to tabulate or visually display results of individual studies and syntheses. | Line 212-214, 229-230 |
|  | 13d | Describe any methods used to synthesize results and provide a rationale for the choice(s). If meta-analysis was performed, describe the model(s), method(s) to identify the presence and extent of statistical heterogeneity, and software package(s) used. | Line 204-214 |
|  | 13e | Describe any methods used to explore possible causes of heterogeneity among study results (e.g. subgroup analysis, meta-regression). | Line 235-242 |
|  | 13f | Describe any sensitivity analyses conducted to assess robustness of the synthesized results. | Line 210-211 |
| Reporting bias assessment | 14 | Describe any methods used to assess risk of bias due to missing results in a synthesis (arising from reporting biases). | Line 200-201 |
| Certainty assessment | 15 | Describe any methods used to assess certainty (or confidence) in the body of evidence for an outcome. | Not Applicable |
| **RESULTS** | | |  |
| Study selection | 16a | Describe the results of the search and selection process, from the number of records identified in the search to the number of studies included in the review, ideally using a flow diagram. | Line 217-221 |
|  | 16b | Cite studies that might appear to meet the inclusion criteria, but which were excluded, and explain why they were excluded. | Line 157-158 |
| Study characteristics | 17 | Cite each included study and present its characteristics. | Line 229-230 |
| Risk of bias in studies | 18 | Present assessments of risk of bias for each included study. | Line 284-285 |
| Results of individual studies | 19 | For all outcomes, present, for each study: (a) summary statistics for each group (where appropriate) and (b) an effect estimate and its precision (e.g. confidence/credible interval), ideally using structured tables or plots. | Line 232-242 |
| Results of syntheses | 20a | For each synthesis, briefly summarise the characteristics and risk of bias among contributing studies. | Line 229-230, 284-285 |
|  | 20b | Present results of all statistical syntheses conducted. If meta-analysis was done, present for each the summary estimate and its precision (e.g. confidence/credible interval) and measures of statistical heterogeneity. If comparing groups, describe the direction of the effect. | Line 232-242 |
|  | 20c | Present results of all investigations of possible causes of heterogeneity among study results. | Line 235-237 |
|  | 20d | Present results of all sensitivity analyses conducted to assess the robustness of the synthesized results. | Line 274-281 |
| Reporting biases | 21 | Present assessments of risk of bias due to missing results (arising from reporting biases) for each synthesis assessed. | Line 288-289 |
| Certainty of evidence | 22 | Present assessments of certainty (or confidence) in the body of evidence for each outcome assessed. | Not Applicable |
| **DISCUSSION** | | |  |
| Discussion | 23a | Provide a general interpretation of the results in the context of other evidence. | Line 294-296, |
|  | 23b | Discuss any limitations of the evidence included in the review. | Line 296-300 |
|  | 23c | Discuss any limitations of the review processes used. | Line 333-342 |
|  | 23d | Discuss implications of the results for practice, policy, and future research. | Line 328-330, 344-347 |
| **OTHER INFORMATION** | | |  |
| Registration and protocol | 24a | Provide registration information for the review, including register name and registration number, or state that the review was not registered. | Line 144-145 |
|  | 24b | Indicate where the review protocol can be accessed, or state that a protocol was not prepared. | Line 144-145 |
|  | 24c | Describe and explain any amendments to information provided at registration or in the protocol. | Not Applicable |
| Support | 25 | Describe sources of financial or non-financial support for the review, and the role of the funders or sponsors in the review. | Line 379-380 |
| Competing interests | 26 | Declare any competing interests of review authors. | Line 382-384 |
| Availability of data, code and other materials | 27 | Report which of the following are publicly available and where they can be found: template data collection forms; data extracted from included studies; data used for all analyses; analytic code; any other materials used in the review. | Not Applicable |

*From:*  Page MJ, McKenzie JE, Bossuyt PM, Boutron I, Hoffmann TC, Mulrow CD, et al. The PRISMA 2020 statement: an updated guideline for reporting systematic reviews. BMJ 2021;372:n71. doi: 10.1136/bmj.n1

| **Supplementary Table. 1 Perioperative outcomes** | | | | | | | | | | | |
| --- | --- | --- | --- | --- | --- | --- | --- | --- | --- | --- | --- |
| **Characteristics** | **Andah,**  **2021** | **Salib,**  **2019** | **Ochiai,**  **2023** | **Nessler, 2020** | **Di Martino,**  **2023** | **Goyal,**  **2022** | **Minutillo, 2023** | **Sarpong, 2023** | **Iturregui, 2023** | **Khan,**  **2023** | **Huebschmann, 2024** |
| **Functionality** |  |  |  |  |  |  |  |  |  |  |  |
| **Length of stay, aadays — aamean (range)** |  |  |  | LOS, 2 (0–5) |  |  | LOS: DA 1 (1–2), Pos 2 (2–3) |  |  |  | LOS: DA 2 (0-7), Pos 3 (0-7), Lat 3 (0-7) |
| **Score — aamean (SD)** |  | HHS, Pre: 50 (NR), Post: 81 (28–100) | JOA, Pre: 56 (10), Post: 88 (8) |  |  |  |  |  |  | HOOS-JR*, Pre: 1 – 44 (16), 2 - 45 (16), ≥3 - 44 (12) Post: 1 - 78 (21), 2 - 82 (18), ≥3- 71 (13) |  |
| **Complications —**  **n (%)** | Revision,  0 | Aseptic loosening,  1 (NR);  Infection, 2 (NR);  DVT, 1 (2);  Revision, 4 (NR) | Aseptic loosening, 0;  Infection, 0;  Revision,  2 (3) | Aseptic loosening,  1, (NR);  Infection,  0;  DVT, 1;  Fracture, 1 (NR) | Aseptic loosening,  Stem, 1 (0.2),  Cup, 4 (1);  Infection, 2 (0.5);  Fracture, 2 (0.5);  Revision, 27 (NR) |  |  | Revision,  DAA: 1 (NR),  PL: 1 (NR) | Revision,  DAA: 1 (1),  AL 4 (5),  Pos 4 (4) |  |  |
| **Radiographic,**  **degrees — mean (SD) or (range)** |  |  |  |  |  |  |  |  |  |  |  |
| **Cup** | Anteversion,  26 (NR);  Abduction,  39 (NR) |  | **Anteversion**, dislocators 17 (4) non-dislocators, 17 (6); **Abduction**, dislocators, 44 (9) non-dislocators, 43 (6);  **Malalignment rate****, dislocators 80, non-dislocators 81 | **Anteversion**,  20 (NR);  **Abduction**,  45 (NR) |  | **Anteversion**, DA: Pre: 21 (19–22), Post: 23 (22–24); DL: Pre: 19 (19–20), Post: 22 (21–23); **Abduction**, DA: Pre: 43 (40–45), Post: 42 (41–44); DL: Pre: 43 (41–44), Post: 41 (40–42) |  |  |  |  |  |
| **Spinal-pelvic aaaalignment** | Standing LL,  52 (NR);  PI-LL mismatch,  15 (NR) |  |  |  |  | **Standing LL**, DA: Pre: 36 (31–41), Post: 39 (35–43), DL: Pre: 40 (37–42), Post: 43 (41–46); **PI-LL mismatch**, DA: Pre: 23 (18–28), Post: 29 (14–44), DL: Pre: 19 (16–22), Post: 14 (11–17) |  |  |  |  |  |
| **Sacral** |  |  | **Slope**, Dislocators, 38 (7) non-dislocators, 32 (9); **Tilt**, dislocators, 35 (16) non-dislocators, 24 (11) |  |  | **Slope**, DA: Pre: 33 (30–36), Post: 37 (34–40); DL: Pre: 37 (34–39), Post: 38 (36–40) |  |  |  |  |  |
| **Pelvic** |  |  | **Incidence**, dislocators, 72 (18) non-dislocators, 55 (13) |  |  | **Tilt**, DA: Pre: 25 (22–28), Post: 25 (22–27), DL: Pre: 23 (21–26), Post: 22 (20–24); **Incidence**, DA: Pre: 59 (54–63), Post: 67 (53–82); DL: Pre: 59 (55–62), Post: 57 (55–60) |  |  |  |  |  |

Note. Due to rounding, all percentages may not total 100%. If no approach is specified, values represent the entire cohort. Abbreviations: HHS, Harris Hip Score ;JOA, Japanese Orthopaedic Association score ; LOS, Lenth of stay ; HOOS-JR, Hip Disability and Osteoarthritis Outcome Score for Joint Replacement ; LL, lumbar lordosis; PI-LL, pelvic incidence-lumbar lordosis; Pos, Posterior; Lat, lateral; DA, Direct anterior; AL, Anterolateral; PL, Posterolateral; DL, Direct lateral; DS, Direct superior; NR, not reported. * khan reported values stratified by the number of level fused. ** defined as out of Lewinnek’s safe zone, reported as percent.

**Supplementary Table. 2 Risk of bias summary for non-randomized studies (ROBINS-I)**

| **Study** | **Bias due to confounding** | **Bias in selection of participants** | **Bias in classification of interventions** | **Bias due to deviations from intended interventions** | **Bias due to missing data** | **Bias in measurement of outcomes** | **Bias in selection of the reported result** | **Overall risk of bias judgement** |
| --- | --- | --- | --- | --- | --- | --- | --- | --- |
| Andah, 2021 | Serious | Low | Low | Low | Low | Low | Low | Serious |
| Salib, 2019 | Moderate | Low | Low | Low | Low | Low | Low | Moderate |
| Ochiai, 2023 | Serious | Low | Low | Low | Low | Low | Low | Serious |
| Nessler, 2020 | Serious | Low | Low | Low | Low | Low | Low | Serious |
| Goyal, 2022 | Serious | Low | Low | Low | Low | Low | Low | Serious |
| Minutillo, 2023 | Moderate | Low | Low | Low | Low | Low | Low | Moderate |
| Sarpong, 2023 | Moderate | Low | Low | Low | Low | Low | Low | Moderate |
| Iturregui, 2023 | Moderate | Low | Low | Low | Low | Low | Low | Moderate |
| Di Martino, 2023 | Serious | Low | Low | Low | Low | Low | Low | Serious |
| Khan, 2023 | Serious | Low | Low | Low | Low | Low | Low | Serious |
| Huebschmann, 2024 | Serious | Low | Low | Low | Low | Low | Low | Serious |

**Supplementary Figures**


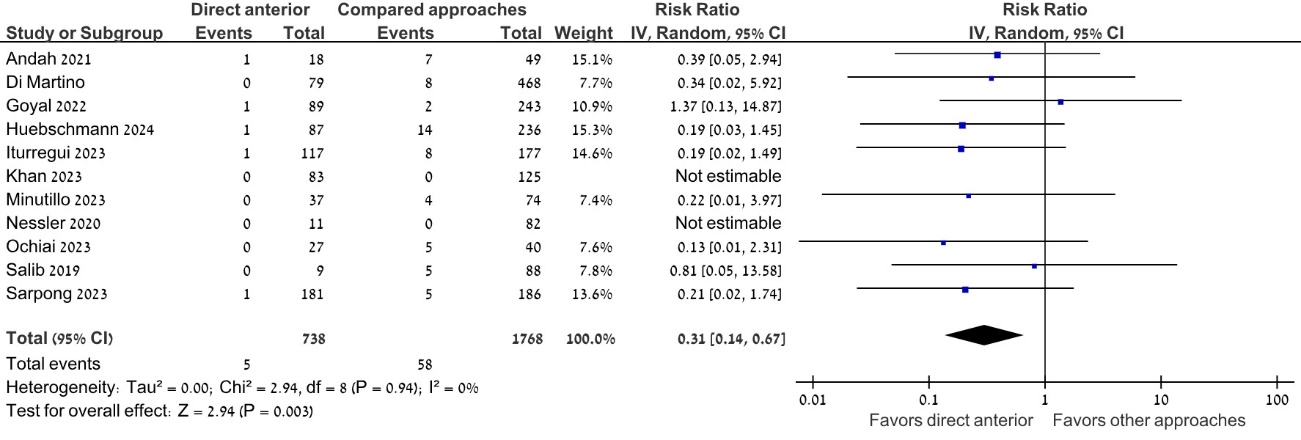


**Supplementary Fig. 1** sensitivity analysis using the generic inverse variance method to assess the relative risk of dislocations via the direct anterior approach compared with other approaches in patients with stiff lumbar spine.


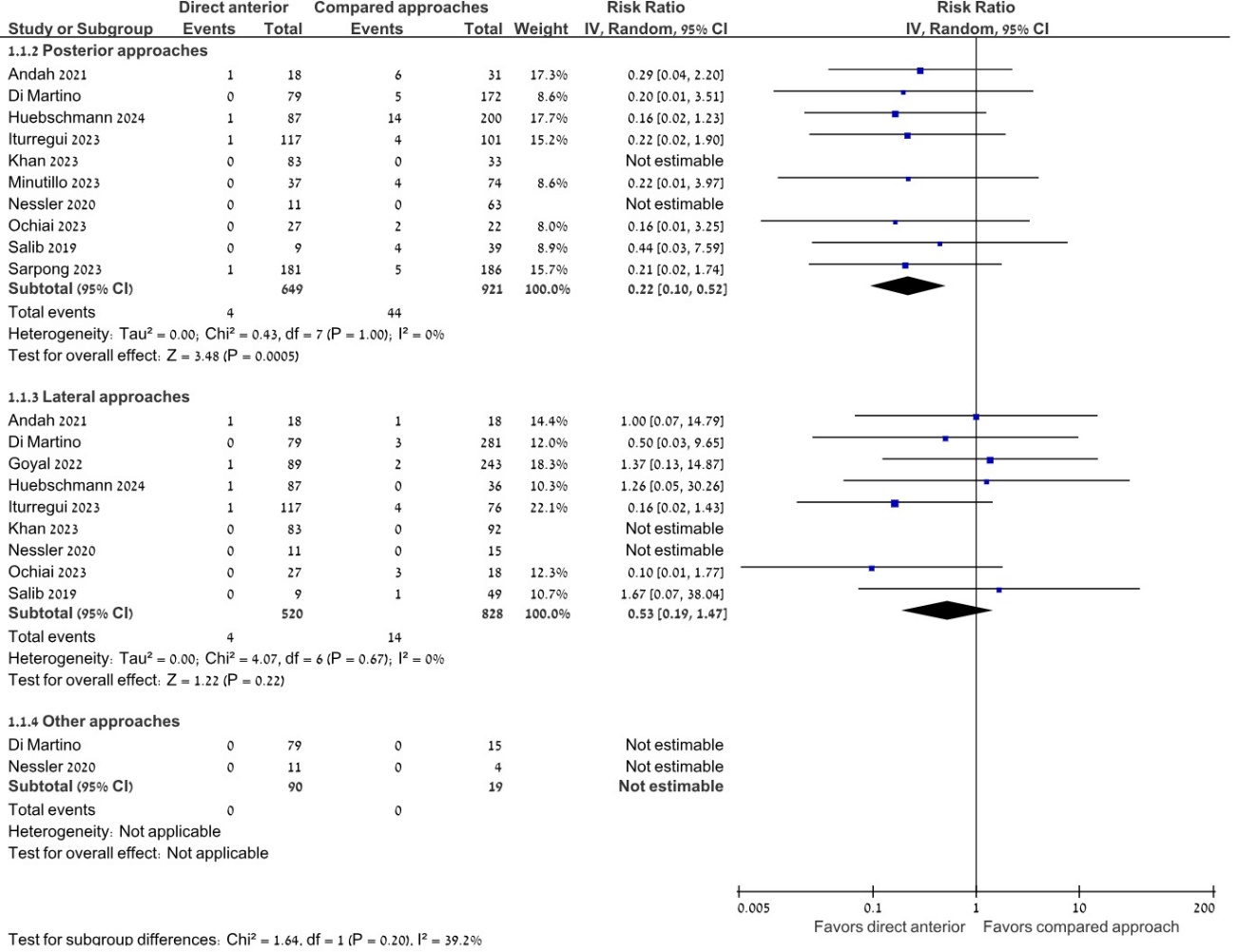


**Supplementary Fig. 2** sensitivity sub-group analysis using the generic inverse variance method to assess the relative risk of dislocations via the direct anterior approach compared with posterior and lateral approaches in patients with stiff lumbar spine


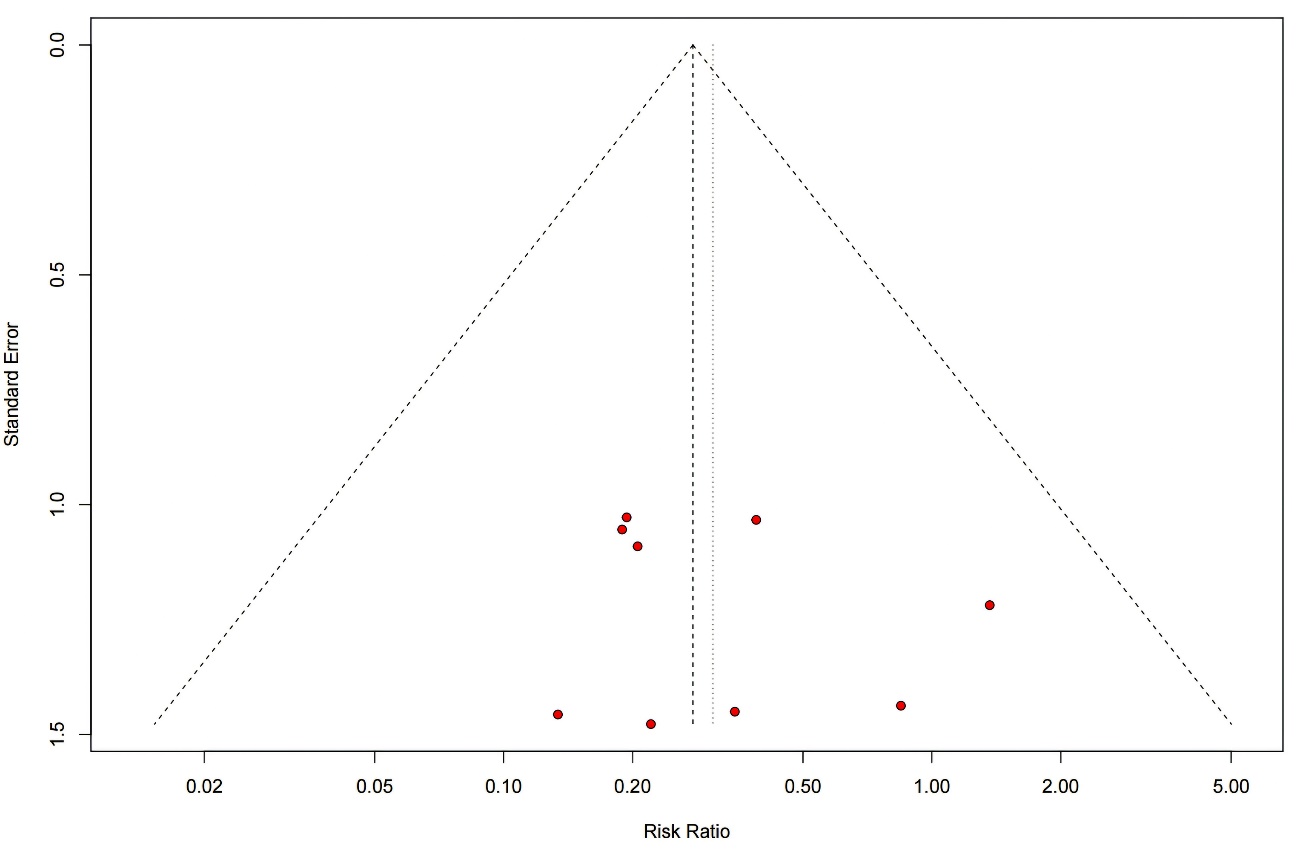


**Supplementary Fig. 3** Funnel plot for the outcome of dislocations.
